# Five-Year Incidence of Primary Open-Angle Glaucoma and Rate of Progression in Health Center-Based Korean Population: The *Gangnam Eye Study*

**DOI:** 10.1371/journal.pone.0114058

**Published:** 2014-12-04

**Authors:** Young Kook Kim, Hyuk Jin Choi, Jin Wook Jeoung, Ki Ho Park, Dong Myung Kim

**Affiliations:** 1 Department of Ophthalmology, Jeju National University Hospital, Jeju, Korea; 2 Department of Ophthalmology, Seoul National University College of Medicine, Seoul, Korea; 3 Healthcare System Gangnam Center, Seoul National University Hospital, Seoul, Korea; 4 Department of Ophthalmology, Seoul National University Hospital, Seoul, Korea; Casey Eye Institute, United States of America

## Abstract

**Objective:**

To investigate the 5-year incidence and progression rate of primary open-angle glaucoma (POAG) in a health-center-based Korean population.

**Methods:**

The study population comprised 5,021 subjects who participated in standardized health screening (including non-contact tonometry and fundus photography) at the Gangnam Healthcare Center during the period from January 2005 to December 2006 and again from January 2010 to December 2011. Among these subjects, 948 (18.9%) with findings suggestive of glaucoma were subjected to a comprehensive glaucoma evaluation, which included applanation tonometry and standard automated perimetry. Based on the results, the subjects were diagnosed as POAG suspect or definite POAG.

**Results:**

The 5-year incidences of POAG suspect and definite POAG were 0.84% (42 subjects) and 0.72% (36 subjects), respectively. The rate of progression from POAG suspect to definite POAG was 4.75% per year. In subjects with a baseline intraocular pressure (IOP) >21 mmHg, the incidence of POAG suspect or definite POAG was significantly higher than in those with a baseline IOP≤21 mmHg (32% vs. 1.05%; *P*<0.001). A multivariate analysis showed that the progression from POAG suspect to definite POAG was significantly associated with older age (odds ratio [OR], 1.07; 95% confidence interval [CI], 1.03–1.10), higher baseline IOP (OR, 1.10; 95% CI, 1.01–1.24), higher body mass index (BMI) (OR, 1.15; 95% CI, 1.03–1.31), higher education level (OR, 1.57; 95% CI, 1.05–2.17), and higher hematocrit level (OR, 1.22; 95% CI, 1.08–1.43).

**Conclusions:**

In the health-center-based Korean population, the 5-year incidence of POAG was 0.72%, and the rate of progression from POAG suspect to definite POAG was 4.75% per year. This study identified old age, high baseline IOP, high BMI, high level of education, and high hematocrit level as significant risk factors for incident POAG.

## Introduction

Glaucoma is the leading cause of irreversible blindness, affecting 66.8 million people worldwide [1], [2]. As the population ages, this figure is expected to increase. Therefore, the epidemiology of glaucoma is of vital importance for establishing appropriate clinical and public health intervention strategies. The epidemiological features of primary open-angle glaucoma (POAG) have been identified in several population-based studies including the Visual Impairment Project in Melbourne, the Rotterdam Study in the Netherlands, the Los Angeles Eye Study in California, and the Barbados Incidence Study of Eye Disease [3]–[6]. Most studies, however, have focused on POAG prevalence, with relatively few investigating the incidence of POAG in longitudinal cohorts. Furthermore, few studies have focused on POAG incidence in Asian populations.

This study was designed to investigate POAG incidence and rate of progression in a health-screened Korean population. The information gained will be helpful for understanding whether or not there are ethnic differences in POAG occurrence, and could help identify risk factors associated with its progression. This large-scale, longitudinal cohort study should facilitate the design of strategies for the prevention of disease in cases with modifiable risk factors.

The *Gangnam Eye Study* is an ongoing cohort study designed in 2004. The study population comprises individuals who had participated in a glaucoma-screening program at the Gangnam Healthcare Center of Seoul National University Hospital. Its primary objective was to evaluate POAG prevalence and incidence during the longitudinal follow-up. The study’s secondary objectives included determining baseline demographics and systemic risk factors, quantifying the diagnostic performance of examination findings, identifying the relevant risk factors of POAG, and elucidating the clinical role of glaucoma-screening strategies.

## Methods

This investigation was based on the *Gangnam Eye Study*, an ongoing cohort study conducted at the Gangnam Healthcare Center at Seoul National University Hospital. It was approved by the Institutional Review Board of Seoul National University Hospital and conducted in accordance with the tenets of the Declaration of Helsinki. Written informed consent was obtained from all subjects.

### Study Population

**Figure 1:**
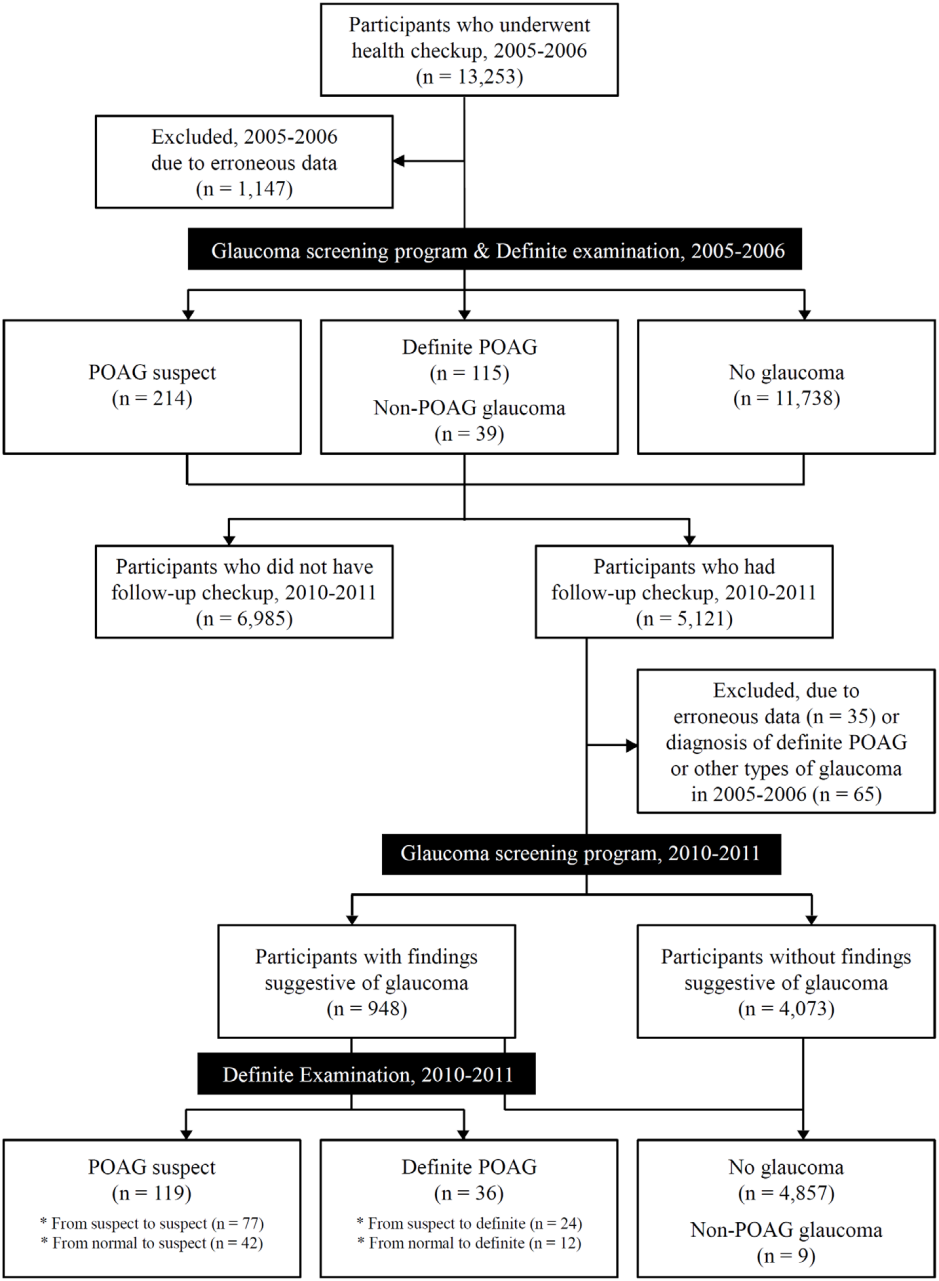
Flow diagram of participant progress in *Gangnam Eye Study*.

This study enrolled individuals who had been attending a healthcare center for general checkups. The inclusion criteria were as follows: participation in a glaucoma-screening program at the Gangnam Healthcare Center of Seoul National University Hospital during the period from January 2005 to December 2006 and then again during the period from January 2010 to December 2011, and age ≥40 years at the time of the first exam (Figure 1). Individuals were excluded if they had a history of intraocular surgery other than uncomplicated cataract surgery or of diseases that could affect the visual field (e.g., diabetic retinopathy, retinal vein occlusion, ischemic optic neuropathy, pituitary lesions, and demyelinating diseases).

### Screening Examination

All of the subjects underwent a default screening test including measurements of body height, body weight, blood pressure (BP), and blood and urine sampling. Also, each subject answered a standard questionnaire, administered by trained research technicians, regarding the presence of systemic diseases (e.g., diabetes mellitus, arterial hypertension, coronary heart disease, asthma, hyperlipidemia). Each participant’s socioeconomic status was evaluated according to their level of education and monthly family income. The level of education was categorized as “primary school education,” “middle school education,” “high school education,” “college education,” or “graduate school education”. The level of monthly family income was categorized as “<$2000 (USD),” “$2000–5000 (USD),” “$5000–8000 (USD),” or “>$8000 (USD)”. Further, the level of smoking was categorized as “have never smoked,” “previously smoked but no longer smoking,” or “currently smoking,” and the level of alcohol consumption was categorized as “do not drink any,” “less than once a month,” “2–3 times a month,” “1–2 times a week,” “3–4 times a week,” or “almost every day”.

### Glaucoma-Screening Program

The glaucoma screening included measurements of intraocular pressure (IOP) and fundus photography. IOP was measured using a non-contact tonometer (model CT10; Topcon Inc., Tokyo, Japan). Fundus photos were taken using a 45° digital non-mydriatic fundus camera (model EOS D60; Canon Inc., Utsunomiya, Japan). Two experienced ophthalmologists (Y.K.K. and H.J.C.), masked to the subjects’ identity, independently evaluated the fundus photographs to check for suspicious findings such as glaucomatous optic neuropathy (GON) or retinal nerve fiber layer (RNFL) defects. The criteria for suspicious findings were as follows:

- Suspected GON: vertical cup-to-disc ratio (VCDR) ≥0.6, VCDR difference ≥0.2 between the eyes, minimal neural rim width <0.1 times the disc diameter, or disc hemorrhage.
- Suspected RNFL defect: width greater than that of a major retinal vessel when measured at the disc edge, diverging in an arcuate or wedge shape.
- Baseline IOP≥22 mmHg [7].

### Definitive Examination

All of the subjects suspected to have glaucoma underwent a comprehensive glaucoma evaluation, including Goldmann applanation tonometry (model AT900; Haag-Streit, Köniz, Switzerland), Goldmann 3-mirror gonioscopy, stereoscopic disc photography, red-free RNFL photography (CF-60Uvi; Canon Inc., Utsunomiya, Japan), and standard automated perimetry (HFA II 720i; Carl Zeiss Meditec Inc., Dublin, CA). GONs and glaucomatous visual field losses (GVFLs) were evaluated by the same physicians. Any disagreements were settled via discussion; when necessary, an additional grader (K.H.P.) was consulted.

### Definitions

The baseline IOP was defined as the IOP measured using a non-contact tonometer during the period from January 2005 to December 2006. For all subjects, glaucomatous optic neuropathy and GVFL were defined based on the following criteria [4]:

- GON: VCDR≥0.7 or VCDR difference ≥0.2 between the eyes, or minimal neural rim width <0.1 times the disc diameter on fundus photos.
- GVFL: ≥2 of the following 3 criteria: (1) the presence of a cluster of 3 points on a pattern deviation probability plot with *P*<0.05, one of which with *P*<0.01; (2) a pattern standard deviation with *P*<0.05; (3) glaucoma hemifield test results outside the normal limits.

Subjects were deemed to have definite POAG if they met both of the following criteria: (1) presence of typical GON with compatible GVFL; (2) normal anterior chamber angles. “POAG suspect” was defined as follows: (1) the presence of typical GON in the absence of compatible GVFL; (2) normal anterior chamber angles. “No glaucoma” was defined as follows: (1) the absence of GON or GVFL; (2) normal anterior chamber angles.

The incidence of POAG suspect was defined as its development during follow-up in at least 1 eye, with no glaucoma present at baseline in either eye. The incidence of definite POAG was defined as its development during follow-up in at least 1 eye, with POAG suspect or no glaucoma present in either eye at baseline. Progression from POAG suspect to definite POAG was defined as the development of the latter during follow-up in at least 1 eye, with POAG suspect in either eye at baseline.

### Statistical Analyses

Statistical analyses were performed using a commercially available statistical software package (SPSS for Windows, version 19.0, IBM-SPSS, Chicago, IL). Chi-square tests were used to compare the proportions. Logistic regression models were utilized to analyze the associations between the incidence of POAG and any continuous or categorical independent variables such as age, gender, or personal history. The data were presented as mean values with standard deviations. All of the *P*-values were two-sided, and were considered significant when <0.05.

## Results

A total of 13,253 subjects aged 40 years or older who had participated in a glaucoma-screening program at the Gangnam Healthcare Center, Seoul National University Hospital, during the period from 2005 to 2006 were enrolled. Of these, 238 subjects (1.7%) with a history of intraocular surgery other than uncomplicated cataract surgery, and 441 subjects (3.3%) with diseases that could affect the visual field, were excluded from further analysis, yielding a sample of 12,574 subjects. Another 468 subjects were excluded from the study owing to incomplete medical records (n = 204) or poor-quality fundus photography (n = 264). Ultimately, 12,106 subjects were included (Figure 1).

During the period from 2010 to 2011, 42.3% (5,121) of the 12,106 subjects were subjected to the same glaucoma-screening program described above. Excluding 35 subjects with incomplete medical records during 2010–2011, and excluding 65 subjects with definite POAG or non-POAG glaucoma (46 with definite POAG, 10 with primary angle-closure glaucoma, 4 with pseudoexofoliation glaucoma, 3 with pigmentary glaucoma, and 2 with uveitic glaucoma) during 2005–2006, 5,021 subjects ultimately were included. Among them, 948 subjects (18.9%) were referred for comprehensive glaucoma evaluations (Figure 1).

When comparing the baseline characteristics between the patients who presented for follow-up during 2010–2011 (n = 5,121) and those who did not (n = 6,985), there were no significant differences in age, sex, IOP, or personal history of diabetes mellitus, arterial hypertension, coronary heart disease, asthma, hyperlipidemia, aspirin use, or anti-coagulant use. Additionally, the baseline prevalences of glaucoma were similar (Table 1).

**Table 1:** Demographic Data of Study Participants.

Characteristics at Baseline in 2005–2006	Participants from2005–2006 and Includedin 2010–2011		Participants from2005–2006 and NotParticipating in 2010–2011		*P*Value
No.	5,121		6,985		
Age (yrs) (mean±SD)	51.35±13.12		53.39±14.01		0.062^a^
Women (%)	39.19		42.55		0.087^b^
Intraocular pressure (mmHg)	13.7±3.1		13.9±2.9		0.231^b^
History of arterial hypertension (%)	14.82		12.66		0.091^b^
History of coronary heart disease (%)	2.26		2.29		0.401^b^
History of asthma (%)	2.78		2.57		0.388^b^
History of hyperlipidemia (%)	5.50		6.14		0.102^b^
History of Aspirin use (%)	8.34		7.78		0.165^b^
History of anti-coagulant use (%)	2.34		2.39		0.222^b^
Prevalence in 2005–2006	%	No.	%	No.	
POAG suspect	1.98	101	1.62	113	0.218^b^
Definite POAG	0.90	46	0.99	69	0.741^b^

SD = standard deviation; POAG = primary open-angle glaucoma.

a Independent-samples t-test.

b Chi-square test.

### Five-Year Incidence of Primary Open-Angle Glaucoma

At 5.1±0.5 years after the baseline screening, 78 subjects (95 eyes) in the study group were newly diagnosed as POAG suspect (42 subjects; 52 eyes) or definite POAG (36 subjects; 43 eyes). Additionally, 9 subjects (13 eyes) were newly diagnosed as non-POAG glaucoma: 6 (10 eyes) with primary angle closure glaucoma, 2 (2 eyes) with pseudoexofoliation glaucoma, and 1 (1 eye) with uveitic glaucoma. The 5-year incidence of POAG suspect was 0.84%, and that of definite POAG, 0.72% (Table 2). The 5-year incidence of primary angle-closure glaucoma was 0.12%, and that of pseudoexofoliative glaucoma, 0.04%.

**Table 2:** Five-Year Incidence of Primary Open-Angle Glaucoma (POAG) Stratified by Age and Gender.

		Normal to POAG Suspect		Normal to Definite POAG		POAG Suspect to Definite POAG	
Age group at Baseline (yrs)	Gender (No.)	No.	5-yearIncidence (%)	No.	5-yearIncidence (%)	No.	5-yearIncidence (%)
40–49	Male (1,380)	10	0.72	3	0.22	5	0.36
	Female (938)	5	0.53	1	0.11	2	0.21
	Combined (2,318)	15	0.65	4	0.17	7	0.30
50–59	Male (1,182)	13	1.10	3	0.25	6	0.51
	Female (796)	5	0.63	2	0.25	2	0.25
	Combined (1,978)	18	0.91	5	0.25	8	0.40
60–69	Male (425)	4	0.94	2	0.47	5	1.15
	Female (207)	2	0.97	0	0.00	3	1.45
	Combined (632)	6	0.95	2	0.32	8	1.27
>70	Male (64)	1	1.56	1	1.56	0	0.00
	Female (29)	2	6.90	0	0.00	1	3.45
	Combined (93)	3	3.23	1	1.08	1	1.08
Total	Male (3,051)	28	0.92	9	0.29	16	0.52
	Female (1,970)	14	0.71	3	0.15	8	0.41
	Combined (5,021)	42	0.84	12	0.24	24	0.48

### Rate of Progression from Primary Open-Angle Glaucoma Suspect to Definite Primary Open-Angle Glaucoma

In the study group, the prevalence of POAG suspects in 2005–2006 was 1.98% (n = 101). Of these, 23.77% (n = 24) had progressed to definite POAG by 2010–2011 (Table 2). The rate of progression from POAG suspect to definite POAG was 4.75% per year.

### Association between Intraocular Pressure and Development of Primary Open-Angle Glaucoma

**Figure 2:**
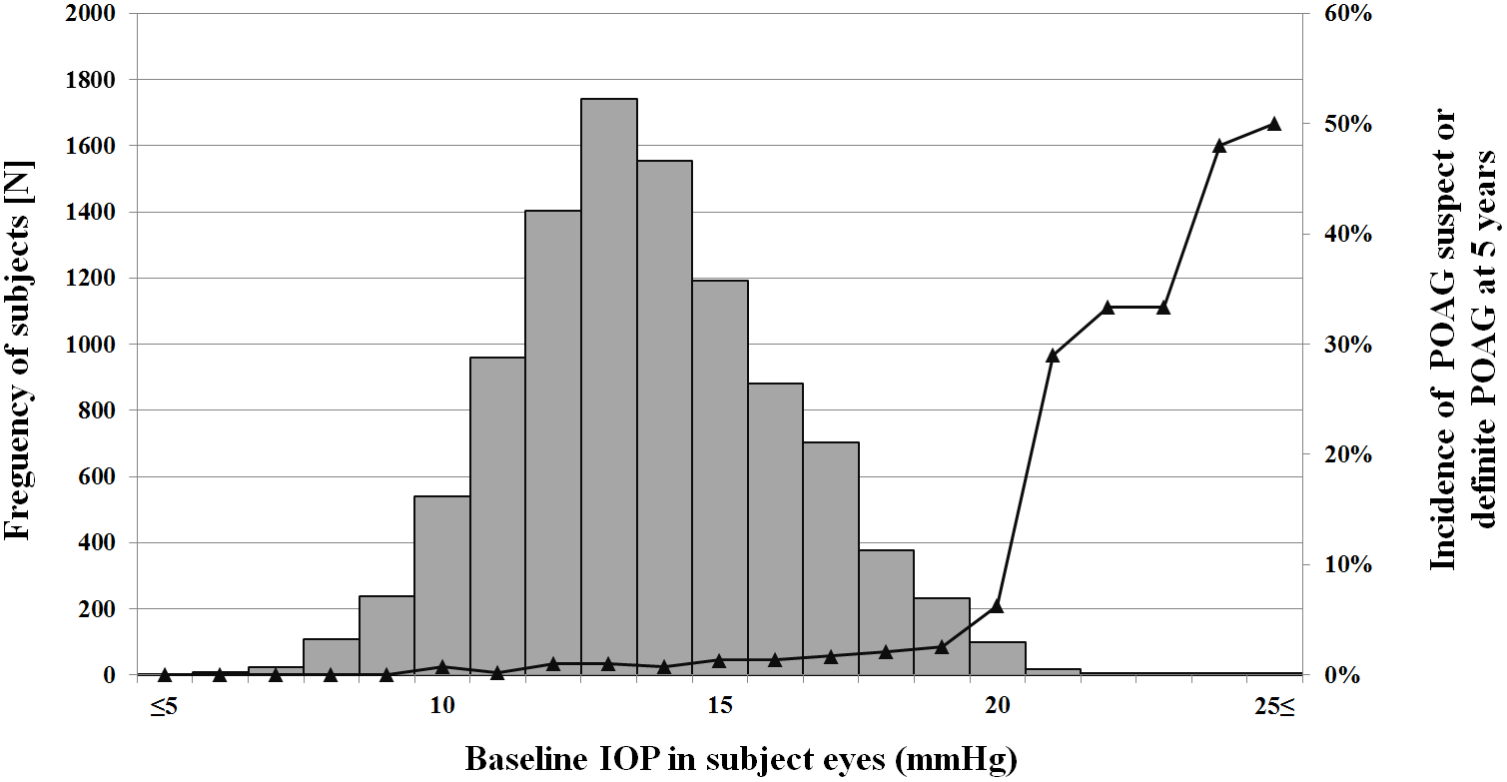
Distribution of baseline intraocular pressure (IOP) and 5-year incidence of primary open-angle glaucoma (POAG) suspect or definite POAG as related to IOP. The bar and broken-line graphs respectively show the distribution of IOP and the 5-year incidence of POAG suspect or definite POAG.

At baseline, the mean IOP in the subject eyes was 13.7±3.1 mmHg (13.6±3.0 mmHg in the right eye; 13.7±3.2 mmHg in the left eye). In the POAG suspect or definite POAG eyes (n = 147), the mean baseline IOP was 16.9±4.3 mmHg, which was greater than that in the eyes without progression (12.8±3.3 mmHg; n = 10,039; *P* = 0.012). In subject eyes with the baseline IOP>21 mmHg (n = 38), the incidence of POAG suspect or definite POAG was significantly higher than in those with the baseline IOP≤21 mmHg (n = 10,096; 32% vs. 1.05%; *P*<0.001) (Figure 2).

### Risk Factors Associated with the Progression of Primary Open-Angle Glaucoma

A univariate analysis showed that the progression from POAG suspect to definite POAG was significantly associated with older age (*P* = 0.021), higher baseline IOP (*P* = 0.023), higher body mass index (BMI) (*P* = 0.006), higher education level (*P* = 0.008), anti-coagulant use (*P* = 0.047), higher hematocrit level (*P* = 0.012), and higher eosinophil level (*P* = 0.048) (Table 3). A stepwise multivariate binary logistic regression analysis included all of the parameters for which the *P*-value of the association with the progression of POAG was ≤0.05 in the univariate analysis. The results revealed that the progression from POAG suspect to definite POAG remained significantly associated with older age (OR, 1.07; 95% CI, 1.03–1.10), higher baseline IOP (OR, 1.10; 95% CI, 1.01–1.24), higher BMI (OR, 1.15; 95% CI, 1.03–1.31), higher education level (OR, 1.57; 95% CI, 1.05–2.17), and higher hematocrit level (OR, 1.22; 95% CI, 1.08–1.43), whereas the associations with anti-coagulant use (*P* = 0.092) and higher eosinophil level (*P* = 0.103) were no longer significant (Table 4).

**Table 3:** Associations Between Glaucoma Progression and Systemic Parameters in Univariate Regression Analysis.

Baseline systemic parameters	Progression from POAG suspect to POAG glaucoma			
	Odds Ratio	95% Cl		*P* Value
Age (years)	1.09	1.05	1.11	0.021
Gender (men/women)	0.79	0.67	0.88	0.789
Intraocular pressure (mmHg)	1.02	1.00	1.05	0.023
Height (mm)	0.98	0.68	1.40	0.975
Body Weight (kg)	1.16	0.92	1.47	0.242
Body Mass Index (kg/m^2^)	1.03	1.01	1.06	0.006
Social history				
Level of Education	1.15	1.07	1.23	0.008
Income (US dollars)	0.94	0.77	1.09	0.722
Smoking (ever-current/never)	0.91	0.46	2.03	0.182
Consumption of alcohol (ever-current/never)	1.55	0.82	2.03	0.521
Personal history				
Diabetes mellitus (present/not present)	0.83	0.67	1.02	0.661
Arterial hypertension (present/not present)	0.42	0.29	0.52	0.454
Coronary heart disease (present/not present)	1.31	1.16	1.46	0.109
Asthma (present/not present)	1.02	0.75	1.41	0.989
Hyperlipidemia (present/not present)	1.31	0.87	1.88	0.128
Aspirin use (yes/no)	2.03	1.21	2.65	0.772
Anti-coagulant use (yes/no)	1.70	1.47	1.97	0.047
Systolic BP (mmHg)	1.45	0.73	2.55	0.485
Diastolic BP (mmHg)	2.00	1.61	2.35	0.089
Blood concentration of				
WBC (per µl)	1.62	0.53	3.14	0.143
Hematocrit (%)	1.17	1.02	1.45	0.012
Eosinophil (%)	1.02	0.83	1.29	0.048
Triglycerides (g/µL)	1.05	0.70	1.49	0.181
HDL (g/dL)	1.56	1.09	2.08	0.840
HBA1C (g/dL)	0.93	0.71	1.32	0.491
Urinary concentration of				
BUN (mg/dL)	0.86	0.42	1.54	0.438
Creatinine (mg/dL)	0.91	0.74	1.92	0.854
Hepatic functions				
ALT (mg/dL)	1.55	0.83	2.88	0.387
AST (mg/dL)	1.01	0.78	1.31	0.467
γ-GT (mg/dL)	1.24	1.03	1.57	0.185
Cancer markers				
AFP (mg/dL)	0.91	0.65	1.31	0.668
CEA (mg/dL)	1.34	0.91	1.78	0.211
Thyroid functions				
TSH (mg/dL)	0.99	0.76	1.20	0.864
Free T4 (mg/dL)	1.51	0.81	2.73	0.392

POAG = primary open-angle glaucoma; CI = confidence interval; BP = blood pressure;

WBC = white blood cell; Hct = hematocrit; HDL = high-density lipoprotein;

BUN = blood urea nitrogen; ALT = alanine aminotransferase; AST = aspartate aminotransferase;

γ-GT = gamma glutamyl transpeptidase; AFP = alpha fetoprotein; CEA = carcinoembryonic antigen; TSH = thyroid-stimulating hormone.

**Table 4:** Associations Between Glaucoma Progression and Systemic Parameters in Multivariate Logistic Regression Analysis.

Baseline systemic parameters	Progression from POAG suspect to definite POAG			
	Odds Ratio	95% Cl		*P*-Value
Age (years)	1.07	1.03	1.10	0.011
Intraocular pressure (mmHg)	1.10	1.01	1.24	0.037
Body Mass Index (kg/m^2^)	1.15	1.03	1.31	0.029
Level of Education	1.57	1.05	2.17	0.017
Anti-coagulant (yes/no)	1.13	0.83	1.43	0.092
Hematocrit (%)	1.22	1.08	1.43	0.008
Eosinophil (%)	1.39	1.12	1.85	0.103

POAG = primary open-angle glaucoma; CI = confidence interval; Hct = hematocrit.

## Discussion

This study investigated the incidence of POAG in an East Asian population. Although there have been numerous epidemiologic studies on glaucoma in comparable populations, the focus in most cases was glaucoma prevalence, not incidence [8]–[11].

Compared with previous population-based studies, this study has several notable strengths: (1) artificial manipulation could be minimized, because the follow-up was performed at the participant’s discretion; (2) various tests could be conducted, including overall physical measurements and check-up examinations on various organs; (3) marked reductions in the cost and the overall time required for the analysis.

This study found that baseline age is a significant risk factor for progression from POAG suspect to definite POAG. Our results suggest that a 1-year difference in age at baseline is associated with a 7% increase in the risk of POAG. Our observations are consistent with previous studies [3], [4], [12], [13]. In the Barbados Incidence Study of Eye Disease and the Rotterdam Eye Study, the risk of developing glaucoma increased 4 and 6%, respectively, in subjects who were 1 year older at baseline [12], [14].

The overall mean baseline IOP of the eyes in this study was 13.7±3.1 mmHg, which is similar to that reported by the Namil study (13.5±2.9 mmHg) [9]. In the POAG suspect or definite POAG eyes, the mean baseline IOP was 16.9±4.3 mmHg, significantly higher than that of the healthy normal eyes (12.8±3.3 mmHg). These results are in agreement with those of the Tajimi study, where IOP was identified as a risk factor for POAG, despite the fact that most (92%) of the POAG patients had IOP≤21 mmHg [15]. Our findings suggest that IOP, even within the normal pressure range, plays a vital role in the pathogenesis of glaucoma. Furthermore, the 5-year incidence of POAG suspect or definite POAG in eyes with the baseline IOP>21 mmHg was significantly higher than in those with the baseline IOP≤21 mmHg (32 vs. 1.05%; P<0.001) (Figure 2). The 5-year incidences of normal-tension/low-tension POAG were 0.51 and 0.20%, respectively.

The present study demonstrated a positive association between BMI and progression of POAG, which finding corresponds well with those of the relevant earlier studies [16], [17]. This connection between obesity and POAG might be related to the presence of excess intraorbital fat tissue, increased episcleral venous pressure, and/or increased blood viscosity with increased resistance to outflow in the episcleral veins [18], [19]. In fact, several previous reports have confirmed the correlation between obesity and increased IOP [20]–[22].

This study also identified high education level as a risk factor for glaucoma progression. However, in the overall analysis, there was no significant association with income, smoking, or consumption of alcohol. These results partially agree with those of previous investigations, such as the Los Angeles Latino Eye Study and the Rotterdam Study, which showed no significant association with education, employment status, income, or insurance status [23], [24]. Nonetheless, considering that a higher level of education is associated with increased myopic refractive error, [25] these factors might yet play a role in the development or progression of glaucoma. Certainly, further study will be necessary to elucidate the complex relationships between socioeconomic status and POAG.

Among the other results of the present study, POAG progression was associated with high hematocrit levels. A previous study attributed development of POAG to blood viscosity [26]. Another study reported that patients with normal-tension glaucoma exhibited abnormal hemorrheological parameters when compared with normal subjects. However, Sekeroglu et al [19]. reported no such differences among patients with POAG, exfoliation glaucoma, or exfoliation syndrome, when compared with normal subjects. Further study will be needed to clarify this issue.

Health screening helps with the early detection of diseases and allows for early access to the proper treatment, which in turn leads to reduced incidence and overall morbidity [27]. In 2011, about 10 million people underwent health screening in Korea. This number is equivalent to 73.7% of all individuals aged ≥40 years. In fact, since the Gangnam Healthcare Center was first established, approximately 400,000 people have presented for checkups, with a yearly average of 41,000 over the last five years. Baseline checkups typically are done 1–5 years later. Among those who presented for health screening during the period from 2005 to 2006, 85.3% presented for more than one follow-up examination. Although the actual follow-up rate in the present study was 42.3%, the cumulative rate of return at any of the 5 years after baseline (85.3%) was similar to the follow-up rates in previous population-based longitudinal studies (76.6–85%) [3]–[5], [28], [29].

In this study, a stepwise glaucoma-screening program was conducted. That is, a general screening (including non-contact tonometry and fundus photography) was initially performed. Based on the results obtained in that general screening, a selective comprehensive glaucoma evaluation was conducted for certain patients. The stepwise screening program followed in this study has several strengths. First, by selecting the subjects who were to undergo comprehensive glaucoma evaluations after the general screening, we were able to reduce the number of unnecessary evaluations. We were also able to detect a considerable number of glaucoma patients without any subjective symptoms. Therefore, stepwise glaucoma-screening strategies (i.e., selective comprehensive glaucoma evaluation following a general screening program) can be useful in clinical and public health interventions.

The *Gangnam Eye Study* eligibility criteria were formulated with the purpose to select subjects at moderate-to-high risk of developing glaucoma. Since numerous previous studies used the “IOP greater than 21 mmHg” criterion for elevated IOP, we used the cut-off of 22 mmHg in the initial screening. If we used only IOP criteria during the screening process, it would have been possible to underestimate the incidence of normal-tension/low-tension POAG. However, since the glaucoma-screening program included optic disc, RNFL and IOP criteria, we are confident that the incidence of POAG, including normal-tension/low-tension POAG, was not underestimated during the screening procedure.

This study has several limitations. First, the study design was not population based, and less than half (42.3%) of the individuals examined at baseline received a follow-up examination. Selection bias might have been operative, because subjects who take care of their own health tend to participate more often than those who do not. Second, the glaucoma-screening program did not include measurements of refractive error or visual field examinations. Particularly, the lack of initial visual field examinations might have resulted in an underestimation of glaucoma prevalence. Third, IOP was measured using a non-contact tonometer during the screening procedure. Only subjects with findings suggestive of glaucoma were offered a comprehensive glaucoma evaluation including Goldmann applanation tonometry. Moreover, the glaucoma-screening program did not include pachymetry measurement. Finally, the study findings cannot be extrapolated to draw conclusions with respect to the prevalence of POAG in any specific region or ethnicity.

In conclusion, in a health-screened Korean population, the 5-year incidence of POAG was 0.72%. Additionally, the rate of progression from POAG suspect to definite POAG was 4.75% per year. This study identified old age, high baseline IOP, high BMI, high level of education, and high hematocrit level as significant risk factors for progression to POAG. It seems clear that stepwise glaucoma-screening strategies can be useful in clinical and public health interventions.
